# Characterization of a Multidrug-Resistant *Salmonella enterica* Serovar Heidelberg Outbreak Strain in Commercial Turkeys: Colonization, Transmission, and Host Transcriptional Response

**DOI:** 10.3389/fvets.2017.00156

**Published:** 2017-09-25

**Authors:** Bradley L. Bearson, Shawn M. D. Bearson, Torey Looft, Guohong Cai, Daniel C. Shippy

**Affiliations:** ^1^National Laboratory for Agriculture and the Environment, United States Department of Agriculture (USDA), ARS, Ames, IA, United States; ^2^National Animal Disease Center, United States Department of Agriculture (USDA), ARS, Ames, IA, United States; ^3^Crop Production and Pest Control Research, United States Department of Agriculture (USDA), ARS, West Lafayette, IN, United States

**Keywords:** *Salmonella enterica* serovar Heidelberg, multidrug-resistant, foodborne outbreak, turkey, colonization, transmission, gene expression

## Abstract

In recent years, multidrug-resistant (MDR) *Salmonella enterica* serovar Heidelberg (*S*. Heidelberg) has been associated with numerous human foodborne illness outbreaks due to consumption of poultry. For example, in 2011, an MDR *S*. Heidelberg outbreak associated with ground turkey sickened 136 individuals and resulted in 1 death. In response to this outbreak, 36 million pounds of ground turkey were recalled, one of the largest meat recalls in U.S. history. To investigate colonization of turkeys with an MDR *S*. Heidelberg strain isolated from the ground turkey outbreak, two turkey trials were performed. In experiment 1, 3-week-old turkeys were inoculated with 10^8^ or 10^10^ CFU of the MDR *S*. Heidelberg isolate, and fecal shedding and tissue colonization were detected following colonization for up to 14 days. Turkey gene expression in response to *S*. Heidelberg exposure revealed 18 genes that were differentially expressed at 2 days following inoculation compared to pre-inoculation. In a second trial, 1-day-old poults were inoculated with 10^4^ CFU of MDR *S*. Heidelberg to monitor transmission of *Salmonella* from inoculated poults (index group) to naive penmates (sentinel group). The transmission of MDR *S*. Heidelberg from index to sentinel poults was efficient with cecum colonization increasing 2 Log10 CFU above the inoculum dose at 9 days post-inoculation. This differed from the 3-week-old poults inoculated with 10^10^ CFU of MDR *S*. Heidelberg in experiment 1 as *Salmonella* fecal shedding and tissue colonization decreased over the 14-day period compared to the inoculum dose. These data suggest that young poults are susceptible to colonization by MDR *S*. Heidelberg, and interventions must target turkeys when they are most vulnerable to prevent *Salmonella* colonization and transmission in the flock. Together, the data support the growing body of literature indicating that *Salmonella* establishes a commensal-like condition in livestock and poultry, contributing to the asymptomatic carrier status of the human foodborne pathogen in our animal food supply.

## Introduction

Food-producing animals such as swine, cattle, and poultry are a major reservoir of the human foodborne pathogen *Salmonella* (1, 2). While some *Salmonella* serovars can cause disease in food-producing animals, most serovars colonize these animals asymptomatically, resulting in the hosts becoming carriers and intermittent shedders of *Salmonella* (1). Poultry (turkey and chicken) are frequent carriers of *Salmonella*, and poultry products represent about 58% of the salmonellosis cases associated with products regulated by the Food Safety Inspection Service (3). The prevalence of foodborne disease outbreaks caused by *Salmonella enterica* serovar Heidelberg (*S*. Heidelberg) has increased over the last decade (4). One of the largest meat recalls in U.S. history resulted from a multistate outbreak of multidrug-resistant (MDR) *S*. Heidelberg in 2011 that caused 136 confirmed cases of human foodborne disease (39% hospitalization rate) and the recall of 36 million pounds of ground turkey meat (5–7). Other recent outbreaks of foodborne illness involving *S*. Heidelberg include contact with dairy bull calves (https://www.cdc.gov/salmonella/heidelberg-11-16/index.html), an international in-flight catered meal (8), and chicken that sickened 634 case patients in 29 states and Puerto Rico (38% hospitalization rate) (9). Analysis of invasive non-typhoidal *Salmonella* isolated in the U.S. from 1996 to 2007 indicated that ~14% of *S*. Heidelberg isolates were from human bloodstream infections with resistance to one or more antimicrobial agents being associated with increased risk for invasive disease (10). *S*. Heidelberg is responsible for 7% of human deaths due to non-typhoidal *Salmonella* in the U.S. (11), the second most frequent serovar causing mortality following serovar Typhimurium. The prevalence of multidrug resistance (resistance to three or more antibiotic classes) in *S*. Heidelberg has increased 2.6-fold since 2004 (12), including resistance to ampicillin, gentamicin, streptomycin, tetracycline, chloramphenicol, kanamycin, and sulfisoxazole (5, 9). Based on 2013 data from the National Antimicrobial Resistance Monitoring System, ~33% of *S*. Heidelberg isolates that cause human foodborne disease are MDR (13).

Understanding the interactions of a foodborne pathogen with its food animal host is important for managing food safety risk; therefore, we investigated the pathogenicity, colonization and transmission potential of an MDR *S*. Heidelberg strain from the 2011 ground turkey outbreak in a natural poultry host—commercial turkeys. The MDR *S*. Heidelberg strain colonized the spleen and tissues of the digestive tract of the turkey without causing noticeable clinical symptoms. Gene expression analysis of blood from 3-week-old turkeys at two days post-inoculation (dpi) suggested only a mild response to the 10^10^ CFU challenge, with 18 genes identified as differentially expressed. In young poults less than one week old, MDR *S*. Heidelberg from inoculated poults was efficiently transmitted to naive poults; these data suggest that young poults are susceptible to colonization by MDR *S*. Heidelberg which may allow for the development of an asymptomatic carrier state in turkeys, thereby confirming this vulnerability as a critical control point to reduce food safety risk in poultry. Collectively, the lack of clinical symptoms and limited gene expression in 3-week-old turkeys in response to the MDR *S*. Heidelberg outbreak strain paired with the efficient transmission, colonization, and proliferation of the strain in newly hatched poults provide insight into potential factors that contribute to the successful colonization of turkey farms with MDR *S*. Heidelberg thereby leading to the recent outbreaks with this human foodborne pathogen.

## Materials and Methods

### Bacterial Strains and Selective Medium

An MDR *S*. Heidelberg strain BSX 126 (2011K-1138; CVM41579) isolated from ground turkey and associated with a 2011 ground turkey outbreak was used for this study (6). Strain BSX 126 is resistant to ampicillin, tetracycline, streptomycin, and gentamicin. In experiment 1 (described below), BSX 126 was inoculated into a turkey, isolated from the spleen at 7 days post-inoculation (dpi) and designated strain SB 395. Growth of *S*. Heidelberg on XLT-4 medium indicated that this serovar is a weak H_2_S producer. Similar to our investigation of *S*. Choleraesuis (14), reducing the tergitol concentration in XLT-4 (Becton, Dickinson and Co., Sparks, MD, USA) to 25% of the normal level allowed *S*. Heidelberg to produce H_2_S, resulting in the visualization of black colonies following 48 h of incubation. Therefore, the bacterial growth medium for culture of *S*. Heidelberg from turkeys was XLT-4 containing 25% tergitol (1.15 ml/l), tetracycline (15 µg/ml), streptomycin (50 µg/ml), and novobiocin (40 µg/ml).

### Animal Trials and Sample Processing

#### Experiment 1

Sixteen 1-day-old tom (male) turkey poults were group housed for two weeks. Fecal samples from the group pen tested negative for *Salmonella* twice using qualitative bacteriology as previously described (15). Turkeys were separated in individual pens and inoculated by oral gavage with 10^8^ (*n* = 8) or 10^10^ (*n* = 7) CFU of MDR *S*. Heidelberg strain BSX 126 at 3 weeks of age. Cloacal temperatures were measured using a Medline thermometer, model # MDS9850B (Mundelein, IL, USA) at 0, 1, 2, and 3 days post-inoculation (dpi). *Salmonella* levels in the feces were determined at 0, 1, 2, 3, 7, 10, and 14 dpi using quantitative and qualitative bacteriology as previously described (16). At 7 dpi, four turkeys from the 10^8^ CFU inoculated group and four turkeys from the 10^10^ CFU inoculated group were euthanized, and tissues [crop, liver, spleen, small intestine (near the cecum), cecum, and cloaca] were collected for *Salmonella* enumeration as previously described (16). At 14 dpi, the remaining turkeys (3–4) were euthanized and evaluated as described earlier.

#### Experiment 2

Thirty-nine 1-day-old tom (male) turkey poults were group housed for the trial. Fecal samples obtained from the shipping crate tested negative for *Salmonella* using qualitative bacteriology. On the day of arrival at NADC, 20 poults (index birds) were inoculated with 2 × 10^4^ CFU SB 395 in 0.25 ml PBS by oral gavage and 19 poults (sentinel birds) received 0.25 ml PBS. At 8 days following MDR *S*. Heidelberg inoculation into the index birds, 10 turkeys from each group (index and sentinel) were euthanized, and the cecum and spleen were collected for *Salmonella* enumeration. The remaining turkeys in both experimental groups were euthanized at 9 days following MDR *S*. Heidelberg inoculation of index birds and the tissues harvested for *Salmonella* enumeration.

### RNA Isolation and Sequencing from Turkey Blood

Using the LeukoLOCK™ Fractionation & Stabilization Kit (ThermoFisher Scientific), blood was collected and fractionated from the wing vein of 3-week-old turkeys (from experiment 1) before inoculation as well as 2 dpi with 10^10^ CFU *S*. Heidelberg following NCAH SOP-ARU-0300. RNA from the leukocyte population (white blood cells) was extracted using the LeukoLOCK™ Total RNA Isolation System (ThermoFisher Scientific). RNA quality and quantity were analyzed on an Agilent 2100 Bioanalyzer (Agilent Technologies, Inc., Santa Clara, CA, USA). Libraries were constructed using the Illumina TruSeq RNA Sample Prep Kit v2 and were sequenced on an Illumina HiSeq 2500 in a 100-cycle paired-end sequencing run (Illumina Inc., San Diego, CA, USA) at the Iowa State University DNA core facility. Sequence data were imported, quality trimmed in CLC Genomic workbench V 9.5.2, and mapped to the *Meleagris gallopavo* reference assembly 5.0 (17). Expression values were calculated only using uniquely mapped reads. Empirical analysis of differential gene expression was performed using the *EdgeR* statistical test, implemented in CLC Genomic workbench, on the raw unique reads (18). Gene expression differences greater than 1.5-fold with false discovery rate-adjusted *P*-values less than 0.05 were considered significant.

## Results and Discussion

### Fecal Shedding, Tissue Colonization, and Transmission of an MDR *S*. Heidelberg Outbreak Strain in Turkeys

**Figure 1:**
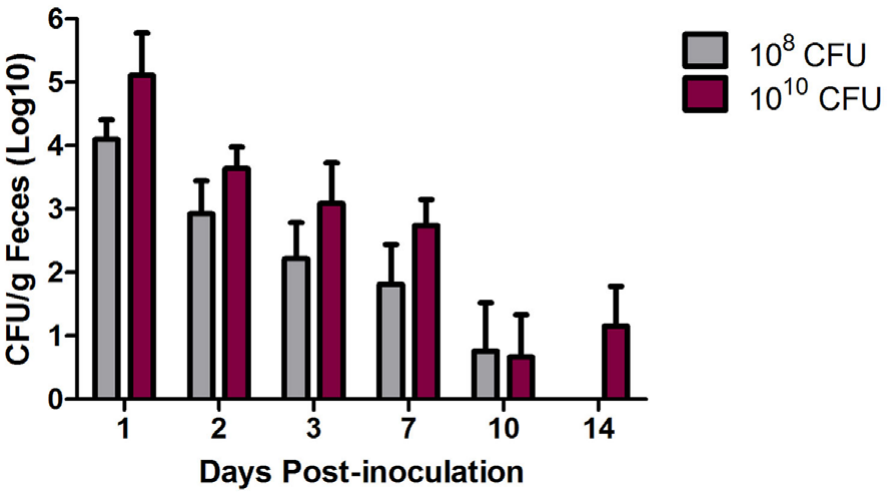
Multidrug-resistant (MDR) *Salmonella enterica* serovar Heidelberg (*S*. Heidelberg) fecal shedding from turkeys. At 3 weeks of age, individually housed turkeys were inoculated with 10^8^ (*n* = 8) or 10^10^ (*n* = 7) CFU of MDR *S*. Heidelberg. Feces was collected at the indicated time points, and quantitative and qualitative bacteriology was performed to determine fecal shedding of MDR *S*. Heidelberg. Four turkeys from each group were euthanized at day 7 dpi resulting in smaller groups for the remaining time points. Error bars indicate SEM.

To evaluate the pathogenicity of an MDR *S*. Heidelberg outbreak strain in turkeys, 3-week-old turkey poults were inoculated with 10^8^ or 10^10^ CFU and monitored for fecal shedding of *Salmonella* and changes in body (cloacal) temperature. Using Bonferroni’s multiple comparison test, no significant difference in average body temperatures at 1, 2, or 3 dpi compared to pre-inoculation was observed at either inoculation dose (data not shown); thus, no fever was induced in the turkeys following *S*. Heidelberg challenge. Fecal shedding of *Salmonella* was detected out to 10 dpi for the 10^8^ CFU inoculated turkeys and to 14 dpi for the 10^10^ CFU inoculated turkeys (Figure 1). During the first week post-inoculation, an ~1-log difference in *Salmonella* shedding between the 10^8^ and 10^10^ CFU inoculated birds was measured, and an ~1-log reduction, regardless of inoculation dose, occurred each day in the turkeys for the first 3 days.

**Figure 2:**
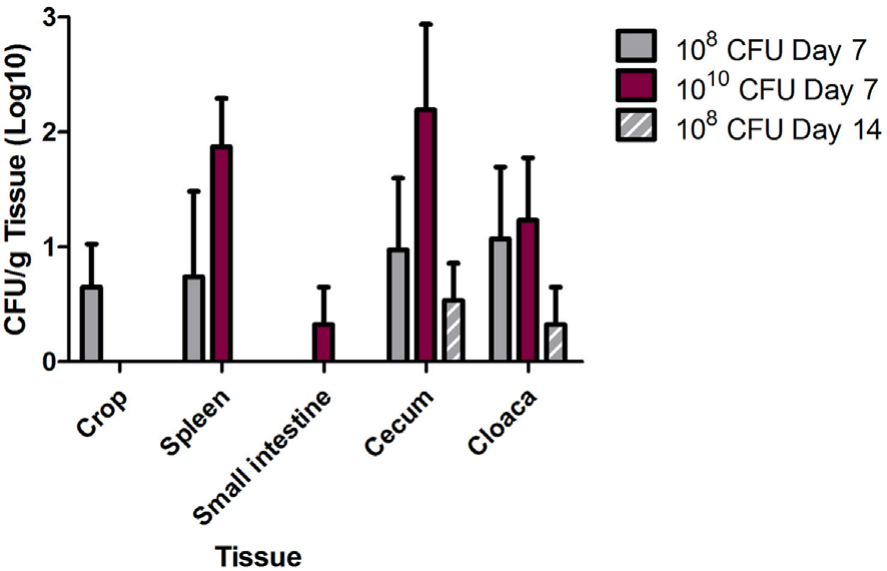
Multidrug-resistant (MDR) *Salmonella enterica* serovar Heidelberg (*S*. Heidelberg) tissue colonization in turkeys. At 3 weeks of age, individually housed turkeys were inoculated with 10^8^ (*n* = 8) or 10^10^ (*n* = 7) CFU of MDR *S*. Heidelberg. At day 7 following *Salmonella* inoculation, four turkeys from each group were euthanized, and the remaining turkeys were euthanized at 14 dpi. Tissues (crop, spleen, small intestine, cecum, and cloaca) were harvested following euthanasia for quantitative and qualitative bacteriology to determine tissue colonization by MDR *S*. Heidelberg. Error bars indicate SEM.

Tissue colonization of *S*. Heidelberg was determined at 7 and 14 dpi for the crop, liver, spleen, small intestine (near the cecum), cecum, and cloaca (Figure 2). At 7 dpi, *S*. Heidelberg was detected in the crop (10^8^ dose; 2/4 birds), spleen (10^8^ and 10^10^ doses; 1/4 and 4/4 birds, respectively), small intestine (10^10^ dose; 1/4 birds), cecum (10^8^ and 10^10^ doses; 2/4 and 3/4 birds, respectively), and cloaca (10^8^ and 10^10^ doses; 2/4 and 3/4 birds, respectively). At 14 dpi, *Salmonella* was only detected in the cecum (2/4 birds) and cloaca (1/4 birds) samples in the turkeys inoculated with *S*. Heidelberg at 10^8^ CFU. *S*. Heidelberg was not detected in the liver at 7 dpi and was therefore not evaluated at 14 dpi.

**Figure 3:**
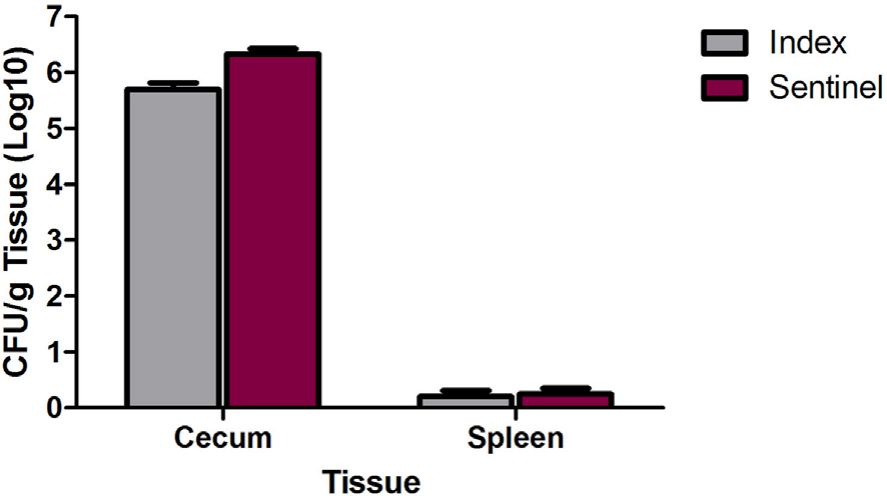
Cecum and spleen colonization by multidrug-resistant (MDR) *Salmonella enterica* serovar Heidelberg (*S*. Heidelberg) in index and sentinel poults. At 1 day of age, 20 poults (index) were directly inoculated with 2 × 10^4^ CFU of MDR *S*. Heidelberg and group housed with 19 poults (sentinel). At days 8 and 9 following index inoculation, 10 poults from each group were euthanized the first day, and the remaining birds were euthanized the second day to harvest tissues. Quantitative and qualitative bacteriology was performed on the cecum and spleen to determine MDR *S*. Heidelberg tissue colonization and *Salmonella* transmission from index to sentinel poults. Error bars indicate SEM.

In a separate study, 1-day-old poults (*n* = 20) were inoculated with 2 × 10^4^ CFU of the MDR *S*. Heidelberg outbreak strain and group housed with mock-inoculated poults (*n* = 19). At 8 and 9 dpi, all poults were euthanized, and spleen and cecal colonization was determined. The spleens from five index and seven sentinel poults were positive for MDR *S*. Heidelberg whereas the cecum in all poults were colonized by *Salmonella* (Figure 3). For the index and sentinel poults, *Salmonella* levels were 5.6 and 6.3 Log10 CFU/g cecum tissue, respectively (Figure 3). Thus, exposure to MDR *S*. Heidelberg earlier in life (1-day-old poults) resulted in higher cecal colonization rates in poults compared to challenge at a later time in life (3 weeks, Figure 2), even with a lower dose of ~10^4^ CFU (compared to the 10^10^ dose at 3 weeks of age). Interestingly, at 8/9 days following oral inoculation of the 1-day-old turkey poults with MDR *S*. Heidelberg, cecum colonization was increased ~2 Log10 CFUs compared to the inoculum dose regardless of whether birds were directly inoculated (index) or following transmission (sentinel) from inoculated poults. In our experience with challenging swine or turkeys with various *Salmonella* serovars, we typically observe a considerable decrease in *Salmonella* CFUs for fecal shedding and tissue colonization by 7 dpi compared to the initial inoculation dose, not an increase as measured in this study. However, our previous experiments were performed with pigs or turkeys that were 3 weeks of age or older. This suggests that the development and maturation of host factors such as immunity and/or the intestinal microbiota play an important role in limiting *Salmonella* colonization in older swine and poultry. Our results with MDR *S*. Heidelberg in turkeys are consistent with an experiment by Menconi et al. who demonstrated that day-of-hatch turkey poults inoculated with ~10^6^ CFU *S*. Heidelberg were colonized with 7.04 and 6.05 Log10 CFU/g cecal contents at 24 and 72 h following inoculation, respectively, with *Salmonella* present in the cecal tonsils of all poults (20/20) at both time points (19). These results indicate that the level of *S*. Heidelberg in the cecal contents of poults at 72 h did not decrease from the inoculum level. The findings of Menconi et al. in turkeys were not replicated in two trials in which day-of-hatch broiler chicks were inoculated with either 10^5^ or 10^6^ CFU of serovar Heidelberg (19); at 72 h following inoculation of chicks with *Salmonella*, the cecal contents were colonized with either 1.08 or 2.96 Log10 CFU *S*. Heidelberg/g tissue. Thus, whereas in turkeys the inoculum dose and the level of cecal content colonization at 72 h were similar, in broiler chicks the colonization level decreased compared to the inoculation dose of *S*. Heidelberg. The authors specifically noted this difference indicating that turkey poults were more susceptible to serovar Heidelberg colonization compared to broiler chicks (19). *S*. Heidelberg colonization of the turkey cecal tonsils and cecal contents could be reduced by inoculating poults with a mixed culture of lactic acid bacteria (LAB) 1 h after *Salmonella* inoculation. The inoculation of poults with LAB reduced *S*. Heidelberg cecal content colonization by ~4 Log10 CFU/g content and cecal tonsil colonization by 55% at 72 h following *Salmonella* inoculation in comparison to inoculation with *S*. Heidelberg alone (19). This supports a role of the turkey intestinal microbiota in limiting colonization of *S*. Heidelberg either due to direct inoculation (e.g., probiotic administration) or maturation of the microbial community with age. The quantity of *S*. Heidelberg in the cecum or cecal tonsils in both our experiment and the trial by Menconi et al. indicates similar levels of colonization (10^6^ CFU), potentially suggesting a threshold for niche colonization in the turkey cecum. Our results further extend the findings of Menconi et al. by demonstrating that in newly hatched turkey poults, *S*. Heidelberg can efficiently colonize the cecum through transmission of the pathogen within the flock. Efficient colonization of young turkey poults may contribute to lifelong colonization of turkeys with *S*. Heidelberg and a human foodborne risk for consumption of turkey meat.

### Transcriptional Response of Commercial Turkeys to an MDR *S*. Heidelberg Outbreak Strain

Gene expression analysis of 3-week-old turkeys in response to an MDR *S*. Heidelberg outbreak strain was conducted by RNA-Seq using total RNA isolated from peripheral blood before and 2 days after inoculation (day 2/day 0). Eighteen genes were differentially expressed at 2 dpi compared to pre-inoculation (Table 1). The expression of three genes was significantly upregulated (*RUFY3, LOC104911311*, and *SERPINB10*). The gene ontology biological process annotation of RUFY3 suggests a role in positive regulation of cell migration; overexpression of RUFY3 caused the formation of F-actin-protrusive structures (invadopodia) and the induction of migration and invasion in human gastric cancer cell line SGC-7901 (20). The predicted gene description for LOC104911311 is “cytokine receptor common subunit beta-like.” SERPINB10 (a.k.a. PI10, bomapin) is a member of the superfamily of serine proteinase inhibitors (serpins) that are key regulators in biological processes ranging from complement activation, coagulation, cellular differentiation, tumor suppression, apoptosis, and cell motility (21). A study by Schleef and Chuang described a role for PI10 in the inhibition of tumor necrosis factor alpha-induced cell death (22).

**Table 1:** Differentially expressed turkey genes in response to multidrug-resistant *Salmonella enterica* serovar Heidelberg (2 dpi/0 dpi).

Gene symbol	Gene description	Fold change	False discovery rate-adjusted *P*-value	Ensembl^a^	Source
*RUFY3*	RUN and FYVE domain containing 3	2.36	3.4957E−05	09878	HGNC Symbol; Acc:HGNC:30285
*LOC104911311*	Predicted: cytokine receptor common subunit beta-like	1.94	4.50681E−05		RefSeq: XM_019611421
*SERPINB1*	Serpin family B member 10	1.81	0.000464941	04373	HGNC Symbol; Acc:HGNC:8942
*CETN3*	Centrin 3	−1.73	−3.35822E−05		RefSeq: XM_010726066
*LOC100545668*	Aldose reductase-like	−1.76	−8.46444E−05	13513	RefSeq: XM_003202470
*UROS*	Uroporphyrinogen III synthase	−1.80	−4.95335E−05	11725	HGNC Symbol; Acc:HGNC:12592
*TTC19*	Tetratricopeptide repeat domain 19	−1.86	−3.24345E−05	06277	HGNC Symbol; Acc:HGNC:26006
*ABCB10*	ATP binding cassette subfamily B member 10	−1.88	−0.000176598		RefSeq: XM_010707019
*GSTA3*	Glutathione S-transferase Alpha 3	−1.94	−0.000161676	13935	UniProtKB/TrEMBL; Acc: D4N2R6
*LOC100547913*	Aquaporin-3	−2.04	−0.000245187	01744	RefSeq: XM_010725198
*CMBL*	Carboxymethylenebutenolidase homolog	−2.08	−6.24874E−05	06110	HGNC Symbol; Acc:HGNC:25090
*FAM207A*	Family with sequence similarity 207 member A	−2.09	−0.000101129		RefSeq: XM_010713129
*UROD*	Uroporphyrinogen decarboxylase	−2.25	−5.47793E−05	10326	HGNC Symbol; Acc:HGNC:12591
*ODC1*	Ornithine decarboxylase 1	−2.36	−0.000127647	14011	HGNC Symbol; Acc:HGNC:8109
*DYX1C1*	Dyslexia susceptibility 1 candidate 1	−4.39	−1.13695E−06	05949	HGNC Symbol; Acc:HGNC:21493
*SCG3*	Secretogranin III	−7.55	−2.57004E−05	06516	HGNC Symbol; Acc:HGNC:13707
*KCNAB1*	Potassium voltage-gated channel subfamily A member regulatory beta subunit 1	−9.43	−7.36884E−06	10715	HGNC Symbol; Acc:HGNC:6228
*DAAM2*	Disheveled associated activator of morphogenesis 2	−12.53	−6.76183E−07	10544	HGNC Symbol; Acc:HGNC:18143

*^a^The Ensembl number is proceeded by ENSSSCG000000*.

Fifteen genes were significantly downregulated in response to MDR *S*. Heidelberg challenge. A range of predicted functions for these genes includes ABC transporter (*ABCB10*), heme biosynthetic pathway (*UROD, UROS*), glutathione transferase (*GSTA3*), polyamine biosynthesis pathway (*ODC1*), calcium binding (*CETN3*), respiratory chain (*TTC19*), and voltage-gated potassium channel activity (*KCNAB1*). *LOC100547913* is predicted to encode aquaporin-3 (AQP3). Aquaporins are involved in transepithelial fluid transport and have been implicated in cell migration by a mechanism that facilitates water transport in lamellipodia of migrating cells (23). In this regard, AQP3 has been associated with macrophage immune function *via* a cellular mechanism involving water and glycerol transport that results in subsequent phagocytic and migration activity (24). AQP3^−/−^ mice had an impaired mucosal innate immune response to *Citrobacter rodentium*, as demonstrated by reduced crypt hyperplasia, decreased epithelial expression of IL-6 and TNF-α, and diminished bacterial clearance (25). If LOC100547913 is AQP3, downregulation of the gene may contribute to the limited immune response observed in the turkeys. The gene with the greatest reduction in expression was *DAAM2*, predicted to encode a key effector in the canonical Wnt signal transduction pathway involved in gene expression regulation during embryonic development and regenerative myelination (26).

Similar to our results, a study of the chicken response to *S. enterica* serovar Enteritidis identified *SERPIN B* as upregulated and *AQP8* (an aquaporin) as downregulated (27). However, the number of differentially expressed genes in the MDR *S*. Heidelberg-challenged turkeys seemed minimal compared to other gene expression studies of poultry in response to *Salmonella* (28). Moreover, a recent study by our group profiling the transcriptome of 3-week-old commercial turkeys in response to *S*. Typhimurium challenge identified over 1,000 differentially regulated genes (manuscript in preparation). In a comparison of the gene expression changes in response to the two *Salmonella* serovars, 17 of the 18 genes differentially expressed in turkeys following *S*. Heidelberg challenge were similarly differentially expressed in response to *S*. Typhimurium challenge (data not shown). Taken together, this MDR *S*. Heidelberg outbreak strain appears capable of colonizing turkeys without inducing a strong host response (transcriptionally or clinically), conceivably due to the commensal-like state of this human foodborne pathogen in turkeys.

In summary, *S*. Heidelberg has been isolated from most food-producing animals, shown increased resistance to antimicrobial agents, and is among the top 5 serovars associated with human foodborne illness (29). Understanding the commensal state established by *S*. Heidelberg (and other *Salmonella* serovars) in livestock and poultry requires investigating the complex interactions of the virulence mechanisms of the particular *Salmonella* serovar and the host’s response to not only initial colonization but also the subsequent establishment of a persistently colonized condition. Numerous host factors play a role in this response including animal genetics, age of exposure, health and immune status, farm husbandry practices, and the microbial composition of the gastrointestinal tract. Our colonization data provide insight into the ability of this serovar to effectively evade host response systems, because the 3-week-old turkeys appeared unaffected by the inoculation with 10 billion *Salmonella* Heidelberg, both clinically and transcriptionally. Furthermore, the efficient transmission, colonization, and proliferation of MDR *S*. Heidelberg from index to sentinel poults during the first week of life suggests that limiting the introduction of *Salmonella* into turkey flocks during the establishment of the intestinal microbiota is critical for control of this human foodborne pathogen. Efficient colonization of turkeys at a young age by serovar Heidelberg may help explain the prevalence of this serovar in human foodborne disease including outbreaks.

## Ethics Statement

Procedures involving animals followed humane protocols as approved by the USDA, ARS, National Animal Disease Center Animal Care and Use Committee in strict accordance with the recommendations in the Guide for the Care, and Use of Laboratory Animals by the National Research Council of the National Academies.

## Author Contributions

BB and SB designed the experiments; BB, SB, and DS were involved in acquisition of the experimental data. BB, SB, TL, GC, and DS performed data analysis and interpretation. The manuscript was drafted and revised for important intellectual content by BB, SB, TL, GC, and DS, as well as, final approval of the version to be published with agreement to be accountable for all aspects of the work in ensuring that questions related to the accuracy or integrity of any part of the work are appropriately investigated and resolved.

## Disclaimer

Mention of trade names or commercial products in this article is solely for the purpose of providing specific information and does not imply recommendations or endorsement by the U.S. Department of Agriculture. USDA is an equal opportunity provider and employer.

## Conflict of Interest Statement

The authors declare that the research was conducted in the absence of any commercial or financial relationships that could be construed as a potential conflict of interest. The reviewer HH declared a shared affiliation, with no collaboration, with the authors to the handling editor.
